# Retraction: Liu, T., et al. Preparation of Amidoxime-Functionalized β-Cyclodextrin-Graft-(Maleic Anhydride-*co*-Acrylonitrule) Copolymer and Evaluation of the Adsorption and Regeneration Properties of Uranium. *Polymers* 2018, *10*, 236

**DOI:** 10.3390/polym12122782

**Published:** 2020-11-25

**Authors:** Liu Yang, Lei Bi, Zhiwei Lei, Yu Miao, Bolin Li, Tonghuan Liu, Wangsuo Wu

**Affiliations:** Radiochemistry Laboratory, School of Nuclear Science and Technology, Lanzhou University, Lanzhou 730000, China; yangl2015@lzu.edu.cn (L.Y.); bil16@lzu.edu.cn (L.B.); leizhw17@lzu.edu.cn (Z.L.); miuy14@lzu.edu.cn (Y.M.); libl15@lzu.edu.cn (B.L.); wuws@lzu.edu.cn (W.W.)

As the authors of the title paper [1], it is with great regret that we inform the readership of *Polymers* that we have asked the journal’s publisher, MDPI, to retract our paper from the scientific literature. Our reason for this action is that we made a mistake inserting a previous experiment result in Table 1, the Raw Ratio of Monomers should be the original we added in the reaction. We want to check it, but we have not been able to find the original data that should have been used for Table 1. We also considered generating a new data set for Table 1, which would take a long time to synthesize. We will re-synthesize the material and character it again, but it will need more time. So we wish to apologize to the Editors, to MDPI, and of course to the wider scientific community, for any inconvenience this action may cause.
